# Atrial Natriuretic Peptide Regulates Ca^2+^ Channel in Early Developmental Cardiomyocytes

**DOI:** 10.1371/journal.pone.0008847

**Published:** 2010-01-22

**Authors:** Lin Miao, Min Wang, Wen-Xuan Yin, Qi Yuan, Ying-Xiao Chen, Bernd Fleischmann, Jürgen Hescheler, Guangju Ji

**Affiliations:** 1 National Laboratory of Biomacromolecules, Institute of Biophysics, Chinese Academy of Sciences, Beijing, China; 2 Institute of Physiology, University of Bonn, Bonn, Germany; 3 Institute of Neurophysiology, University of Cologne, Köln, Germany; Istituto Dermopatico dell'Immacolata, Italy

## Abstract

**Background:**

Cardiomyocytes derived from murine embryonic stem (ES) cells possess various membrane currents and signaling cascades link to that of embryonic hearts. The role of atrial natriuretic peptide (ANP) in regulation of membrane potentials and Ca^2+^ currents has not been investigated in developmental cardiomyocytes.

**Methodology/Principal Findings:**

We investigated the role of ANP in regulating L-type Ca^2+^ channel current (I_CaL_) in different developmental stages of cardiomyocytes derived from ES cells. ANP decreased the frequency of action potentials (APs) in early developmental stage (EDS) cardiomyocytes, embryonic bodies (EB) as well as whole embryo hearts. ANP exerted an inhibitory effect on basal I_CaL_ in about 70% EDS cardiomyocytes tested but only in about 30% late developmental stage (LDS) cells. However, after stimulation of I_CaL_ by isoproterenol (ISO) in LDS cells, ANP inhibited the response in about 70% cells. The depression of I_CaL_ induced by ANP was not affected by either Nω, Nitro-L-Arginine methyl ester (L-NAME), a nitric oxide synthetase (NOS) inhibitor, or KT5823, a cGMP-dependent protein kinase (PKG) selective inhibitor, in either EDS and LDS cells; whereas depression of I_CaL_ by ANP was entirely abolished by erythro-9-(2-Hydroxy-3-nonyl) adenine (EHNA), a selective inhibitor of type 2 phosphodiesterase(PDE2) in most cells tested.

**Conclusion/Significances:**

Taken together, these results indicate that ANP induced depression of action potentials and I_CaL_ is due to activation of particulate guanylyl cyclase (GC), cGMP production and cGMP-activation of PDE2 mediated depression of adenosine 3′, 5′–cyclic monophophate (cAMP)–cAMP-dependent protein kinase (PKA) in early cardiomyogenesis.

## Introduction

It has long been known that cardiac I_CaL_ is under control of the atrial natriuretic peptide (ANP), a member of class of polypeptides that includes also brain natriuretic peptide (BNP) and C-type natriuretic peptide (CNP) [1], [2]. ANP is known to be ontogenetically expressed very early during development [3] and regulate a variety of physiological processes affecting cardiovascular homeostasis. It is preferentially secreted by atrial myocytes under conditions of tachycardia [2], [4]. The regulation through its receptors -ANPR-A and ANPR-B- which are part of a membrane-bound guanylyl cyclase complex and a clearance receptor (ANPR-C) [5], [6] has attracted much attention among cardiac physiologists. Recent studies demonstrate that the genes for all three natriuretic peptide receptor subtypes are expressed in human heart during development [7] as well as in ES cell derived cardiomyocytes [8]. ANP is known to act through direct stimulation of the particulate guanylyl cyclase/cGMP- as well as through a G protein mediated inhibition of the adenylyl cyclase/cAMP system [9]–[11]. However, it still remains under debate as to whether and how ANP modulates chronotropy and inotropy of the heart [12]. Several studies reported the negative inotropic effect induced by ANP, related to intracellular production of cGMP, activating PKG and altering Ca2+ channels to decrease intracellular transients [13]–[15]. However, Lainchbury et al. [16] reported a positive inotropic effect of ANP. ANP is known to depress I_CaL_, however the mechanism of this modulation is still controversial. Gisbert & Fischmeister [17] reported that ANP decreased β-adrenergic agonist pre-stimulated I_CaL_ and had only a negligible effects on basal I_CaL_ in frog isolated cardiac cells. While, Tohse et al [18] detected inhibition of the basal I_CaL_ by ANP *via* production of cGMP and activation of PKG in guinea pig cardiomyocytes. An ANP induced decrease of the basal I_CaL_ was also reported for guinea pig [19], rat [20], chick embryos, human atrial and rabbit heart cells [17], [21]–[23]. Moreover, some authors [24] reported that ANP decreased both basal and cAMP pre-stimulated I_CaL_ in fetal heart cells and increased I_CaL_ in human atrial cells [25]. However, the role of ANP in regulating I_CaL_ in early developmental stages of cardiomyocytes has not been observed.

The embryonic stem cell-derived cardiomyocytes have been demonstrated to be a unique tool for functional studies on early cardiomyogenesis [26], [27]. This model of cardiomyocytes expresses all relevant membrane currents and signaling cascades link to embryonic heart. Previous studies also indicated that nitric oxide (NO) is highly expressed in early developmental stages of cardiomyocytes, and that I_CaL_ was regulated and modulated by muscarinic agonists in cardiomyocytes through NO-dependent pathway [26], [27].

In the present study, we investigated the effects of ANP (rat ANP 3–28) on I_CaL_ in cardiomyocytes derived from mouse ES cells as well as isolated myocytes from mouse embryonic hearts. We found that ANP depressed basal I_CaL_ in early developmental stage and ISO pre-stimulated I_CaL_ in late developmental stage cardiomyocytes. The mechanism by which ANP inhibits I_CaL_ involves activation of the pGC/cGMP pathway, and cGMP-stumulation of PDE2 activity, leading to inhibition of the cAMP/PKA pathway.

## Materials and Methods

### Cell Culture and ES Cell Differentiation Procedure

The murine embryonic stem cell line D3 was used throughout this study. Cells were cultivated and differentiated into spontaneously beating cardiomyocytes in Dulbecco's modified Eagle's medium (DMEM) (Serva, Heidelberg. Germany) supplemented with non-essential amino acids, L-glutamine, β-mercaptoethanol (GIBCO BRL GmbH, Germany), and 15% fetal calf serum (FCS) (selected batches of GIBCO BRL) [26]. The D3 cell line was originally established by cultivation of a disaggregated single blastomere of an eight-cell stage 129/ter Sv mouse embryo on feeder layer cells in culture medium additionally supplemented with 5000 IU leukemia inhibiting factor(LIF). About 60 drops (20 µl of cultivation medium containing 400 cells) were placed on the lids of 10 cm petri dishes filled with phosphate-buffered saline (PBS) and cultivated for 2 days. Aggregates were then transferred from the hanging drops into 6 cm non-adhesive bacteriological petri dishes containing 5 ml cultivation medium and were further cultivated for 5 days (‘7 d’). The resulting embryoid bodies (EBs) were separately placed into each well of 24 well-microwell plates coated with gelatin. During further development of the attached EBs cells of endodermal, ectodermal and mesodermal origin were obtained in the outgrowths. First spontaneously beating cardiomyocyte clusters appeared one or two day (7+1/2 d) after plating of EBs in the outer region of the EB outgrowth. Single cardiomyocytes were prepared from beating cell clusters by collagenase (see below).

### Preparation of Single Cardiomyocytes

Single cardiomyocyte-like cells were isolated at distinct developmental stages: 1) early developmental stage (EDS) when first spontaneously contracting clusters of cardiomyocytes appeared (7+1–4 d); 2) late developmental stage (LDS, 7+9–12 d). The following solutions were used (in mmol/L): a) low Ca^2+^ medium: NaCl 120, KCl 5.4, MgSO_4_ 5, Na pyruvate 5, glucose 20, taurine 20, HEPES [N-(2-Hydroxyethyl) piperazine-N′-2-ethanesulfonic acid] 10, pH 6.9 at 24°C (adjusted with NaOH); b) enzyme medium: Low Ca^2+^ medium supplemented with 1 mg/ml collagenase (collagenase B, Boehringer Mannheim) and 30 µmol/L CaCl_2_; and c) KB medium: KCl 85, K_2_HPO_4_ 30, MgSO_4_ 5, EGTA 1, Na_2_ATP 2, Na pyruvate 5, creatine 5, taurine 20, glucose 20, pH 7.2 at 24°C (adjusted with NaOH).

Beating areas of EBs were dissociated using a microscalpel under microscope in a clean air laminar hood. The isolated beating areas were collected and washed in low Ca^2+^ medium for 30 to 60 min at room temperature. Tissue fragments were then incubated in the enzyme medium for 20 min at 37°C. The dissociation of tissue was completed in KB medium by gentle shaking for 20 min and resting for 40 min at room temperature. The isolated cells were resuspended in cultivation medium supplemented with 20% FCS and plated on sterile, gelatin-coated 12×12 glass cover slips and kept in the incubator for 12 to 24 hours at 37°C and 5% CO_2_. During the first 12 hours of incubation, the isolated cardiomyocytes attached to the surface of the glass cover slips and started spontaneous rhythmical contractions. Single embryonic cardiomyocytes were isolated as described previously [28]. All animal procedures described in this study were performed in adherence with the *Guide for the Care and Use of Laboratory Animals* published by the US National Institutes of Health (NIH Publication No. 85-23, revised 1996), with approval from the Institute of Biophysics Committee on Animal Care. Electrophysiological and immunofluorescence investigations were performed on these cardiomyocytes.

### Electrophysiological Measurements

The whole-cell configuration of the patch-clamp technique was used throughout the study on single cardiomyocytes. The glass cover slips with attached isolated single cardiac myocytes were transferred into the recording chamber and continuously superfused with extracellular solution E1 (table 1) for measurement of action potentials and E2 for the measurement of I_CaL_ (table 1). Membrane potentials were recorded in the current clamp mode and membrane currents in the voltage-clamp mode using an Axopatch 200-B amplifier (Axon Instruments, USA). For measurement of I_CaL_, voltage-clamped cells were held at −40 mV in order to inactivate the sodium currents, and trains of 20 ms lasting depolarizing pulses were applied to a test potential of 0 mV at a frequency of 0.2 Hz. Membrane capacitance was determined using the acquisition/analysis software program ISO2 (MFK, Frankfurt, RG). Data were acquired at a sampling rate of 10 kHz, stored on hard disk and analyzed off-line.

**Table 1:** Internal and external solutions used in the study.

Substances	I1 (mM/L)	I2 (mM/L)	E1(mM/L)	E2 (mM/L)
NaCl	0	0	140	120
KCl	50	0	5.4	5
K-aspartate	80	0	0	0
CsCl	0	120	0	0
CaCl_2_	0	0	1.8	1.8
MgCl_2_	1	3	1	1
EGTA	10	10	0	0
HEPES	10	5	10	10
Glucose	0	0	10	0
Mg-ATP	3	5	0	0
TEA*	0	0	0	20
Na-GTP	0	0	0.42	0

I: internal solution; E: external solution.

* TEA indicates: Tetraethylammonium.

pH: I1 was 7.50 adjusted with KOH, I2 was 7.50 adjusted with CsOH at 23°C. E1 and E2 were 7.40 adjusted with NaOH and TEAOH at 23°C, respectively.

EB and whole embryonic heart action potentials were monitored with conventional 3 M KCl-filled glass microelectrodes (10–20 MΩ) attached to a VF-1 preamplifier (World Precision Instruments, Sarasota, FL).

### Pipettes and Solutions

The solutions used throughout the study are listed in Table 1. The patch pipettes (2–4 MΩ resistance when filled with the internal solution) were pulled from Hilgenberg (FRG) or Clark (England) borosilicate glass capillaries using a Zeitz puller (DMZ, Munich, FRG) and filled with the solution I1 for the measurement of APs. The internal solution I2 was used for the measurement of I_CaL_. Cs^+^ (in internal solution) and tetraethylammonium (TEA, in external solution) were used to block most K^+^ currents and 4-Aminopyridine was added to the extracellular solution to minimize the interference from the transient outward K^+^ current (I_to_). To exclude the possible contamination of I_CaL_ by Na-channel current TTX(10 µM) was added in external solution. The pH of all solutions was adjusted to 7.4 at a temperature of 37°C. Cells were constantly superfused using a gravitational perfusion system, the perfusion rate being approximately 2 ml/min. The chamber volume was 0.5 ml. The temperature of the bath as well as of the perfusion solutions was kept constant at 37°C.

### RT-PCR and Immunofluorescence Study of ANP

Single cell preparations of EDS cardiomyocytes isolated from embryo heart were used for the immunocytochemical investigation of ANP and α-MHC staining. Single cell preparations were fixed in 4% paraformaldehyde in 0.1 M PBS for 20 minutes. Fixed samples were washed three times with PBS (pH 7.4). The cells were incubated with 0.1% triton X-100 for 10 min, followed by incubation with α-MHC antibody (goat anti mouse IgG) and ANP antibody (rabbit anti mouse IgG) in 1% BSA PBS for 2 hr and wash three times at room temperature. Thereafter, cell preparations were then indirectly immunolabelled with a dilution 1∶400 α-MHC donkey anti-goat antibody (labeled with Rhodamine Red-X) and ANP bovine anti-rabbit antibody (labeled with FTTC) for one and half hr at room temperature.

RT-PCR was performed by routine methods on embryo cardiomyocytes dissected from different developmental stages, i.e. E8.5d, E12.5 and E15.5 day, using the following specific primers (forward and reverse, respectively):CCTGTGTACAGTGCGGTGTC, AAGCTGTTGCAGCCTAGTCC.

### Reagents

ANP(3–28,1–28), Met (o)^12^-ANP, ATP-γ-S and forskolin were purchased from Sigma; KT5823 and EHNA were from Calbiochem. All other substances used in the study were from Sigma. ANP 3–28 was used throughout the study, indicated,otherwise.

### Analysis of Data

Averaged results are presented as mean ± standard error of the mean (SEM). Statistical analysis was performed using paired and unpaired Student's t-test, when more than one experimental condition was evaluated a Bonferroni correction was performed, p values of <0.05 were considered significant. The straight line connecting control amplitude of peak I_CaL_ prior to ANP application and after wash-out was used as an estimate for I_CaL_ run-down. For calculation of the ANP-dependent I_CaL_ depression the control value was defined as the peak current taken on the line in the same time-point as the maximal inhibition of I_CaL_ by ANP. For calculation of basal I_CaL_ stimulation the control I_CaL_ was taken as the reference value. Current densities are expressed as current amplitude divided by cell capacitance (pA/pF). To indicate that the recording of the currents is stable, we also analyzed the holding currents as indicated by black dots at 0 pA for each experiment. From the holding currents we know that the leakage of the currents is very small, so in the present study the leakage subtraction was not carried out. The results are expressed as means ± SEM. Significant differences between groups were determined by the Student's *t* test.

## Results

### Expression of ANP in Mouse Embryonic Cardiomyocytes

**Figure 1:**
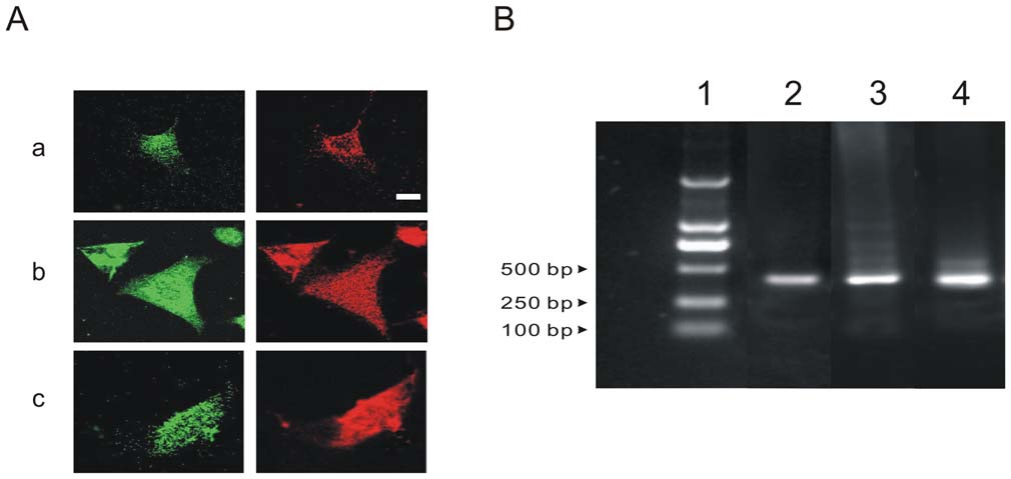
Expression of ANP in mouse embryonic cardiomyocytes. **A**: double staining of ANP (left, green) and α-MHC (right, red) in E8.5d (a), E12.5d (b), and E15.5d (c) cardiomyocytes. **B**: PCR product of ANP. Lanes indicate 1 marker, 2 E8.5d, 3 E12.5d, and 4 E15.5d, respectively. The scale bar is 10 micron.

It has previously been reported that ANP is expressed in cardiomyocytes derived from monkey ES cells [8]. Since nothing was known in mouse embryonic cardiomyocytes, we examined the expression of ANP in this preparation. We first sought to detect the presence of ANP in mouse embryonic cardiomyocytes by double immunofluorescence staining for ANP (green) and α-MHC (red), and the results demonstrated that embryo cardiomyocytes contain ANP in the cytoplasm during all developmental stages (E8.5d, E12.5d, and E15.5d, Fig. 1A). RT-PCR studies also indicated that ANP was expressed at the mRNA level in different developmental stages of mouse embryo heart (Fig. 1B).

### Effect of ANP on Action Potentials in Early Developmental Stage Cardiomyocytes

**Figure 2:**
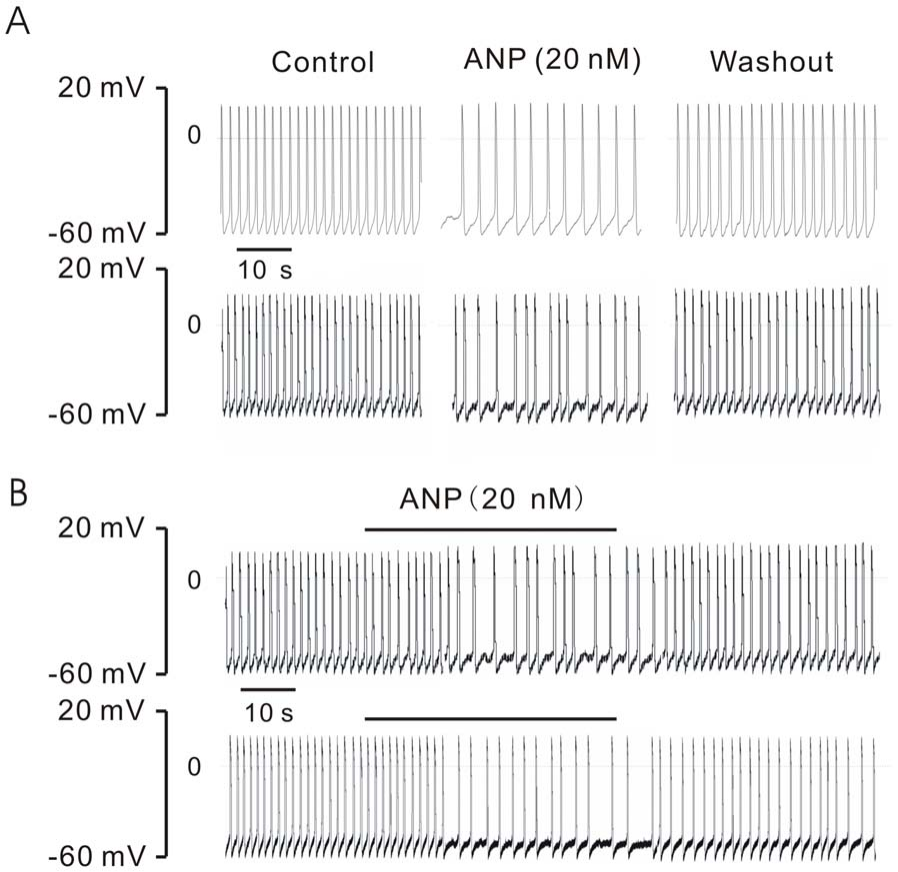
Inhibition of spontaneous action potentials by ANP. **A**: Action potentials recorded from a spontaneously contracting EDS cell (upper) and an E8.5d cardiomyocyte (lower) in the current clamp mode. The frequency of action potentials was decreased by application of ANP in a reversible manner. **B**: action potentials recorded from an early developmental stage EB (upper) and an embryonic 8.5 day heart (lower). As seen in single EDS and E8.5d cells, application of ANP exerted a negative chronotropic effect that was washable.

First evidence for functional effects of ANP on EDS cells was obtained in current clamp experiments. Fast perfusion of spontaneously contracting cardiomyocytes derived from ES cells with 20 nM ANP [18], [23] resulted in a negative chronotropic effect, without concomitant hyperpolarization and this effect was reversible upon washout (Fig. 2A, upper traces). To unequivocally prove the effect of ANP on APs in ES derived cardiomyocytes, the effect of ANP on APs was also examined in cardiomyocytes isolated from early developmental mouse embryonic hearts (E8.5∼10.5d), and similar results as in ES cell- derived cardiomyocyte were obtained, i.e. a reversible decrease in AP frequency in 16 out of 18 experiments (Fig. 2A, lower traces). The effects of ANP were not only seen in single cells but also in integrated preparations, since ANP had also pronounced inhibitory effects on AP frequency in mouse EDS EBs (Fig. 2B, upper traces) and mouse whole embryo heart (E8.5d, Fig. 2B, lower traces). The negative effect of ANP on APs usually was observed about 20 s after fast perfusion in single cardiomyocytes, EBs, and even in E8.5d mouse heart. If the effects of ANP were not washed the APs could be completely inhibited with the time (Fig. S1B).

### ANP Inhibits Basal I_CaL_ in EDS Cardiomyocytes Derived from ES Cells

**Figure 3:**
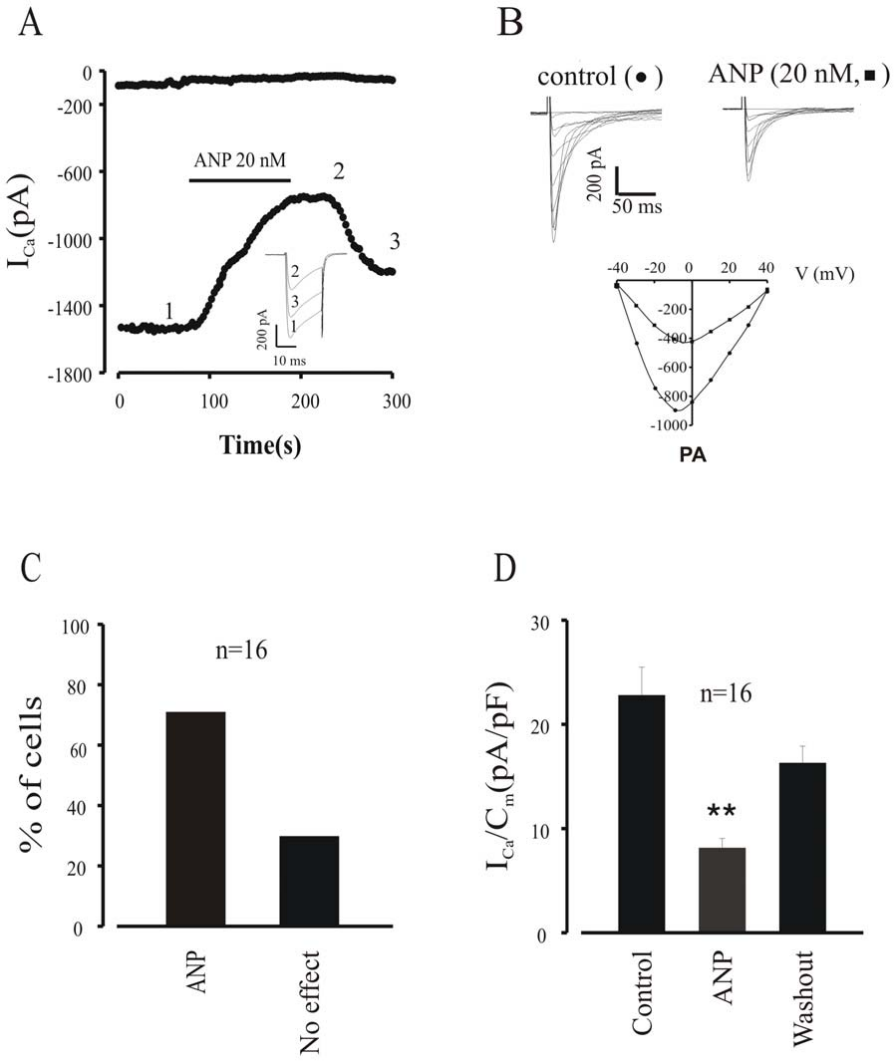
ANP depresses basal I_CaL_ in EDS cardiomyocytes. **A**, Typical recording of I_CaL_ from a single cell. Upon application of 20 nM ANP, rat 3–28, basal I_CaL_ was decreased. This effect could be partially reversed by washout. The inset represents currents recorded as indicated by the mumbers in the time course diagram. **B**, I/V relationship: ANP decreased peak I_CaL_ without a shift in the I/V relationship. **C**, percentage of cells responding to ANP. **D**, density of peak I_CaL_.

The negative chronotropic effect of ANP indicates that ANP must affect the activity of ion channels. Because ANP did not exhibit any effect on I_f_ and I_ks_ (data not shown) we focused our interest on the L-type Ca^2+^ channel current and examined the effect of ANP on I_CaL_ in cardiomyocytes derived from ES cells. The protocols used to measure I_CaL_ was a 20 ms lasting depolarizing pulse to 0 mV from a holding potential of −40 mV (see Materials and Methods). Fig. 3A shows a typical experiment in which 20 nM ANP 3–28 induced a pronounced inhibition of the peak amplitude of I_CaL_. On average (Fig. 3D), ANP induced an inhibition of I_CaL_ by 60.3±10.1% (n = 16, p<0.05) after application of ANP in about 70% cells tested (Fig. 3C), which is similar to our previous study (27). The effect of ANP was always reversible upon washout. The effect of ANP was not voltage-dependent, since ANP induced propotional reduction of peak I_CaL_ at all potentials tested (Fig. 3B). Figure S1 shows concentration-response relation between ANP and the I_CaL._ ANP at concentration above 10 nM decreased the peak current.

It has reported that another type of ANP, ANP 1–28, also has inhibitory effect on I_CaL_ in rabbit heart cells [18]. To examine if this type of ANP has the similar effect on I_CaL_ as ANP 3–28 in our model, equal concentration of ANP1–28 was tested. The results suggested that ANP1–28 (20 nM) had little effect on basal I_CaL_ in EDS cells, but increasing its concentration to 50 nM led to a similar inhibition of I_CaL_ as 20 nM ANP 3–28 (data no shown). As a negative control, the inactive form of ANP, Met (o)^12^-ANP had no effect on I_CaL_ at concentrations ranging from 10 to 100 nM (data not shown).

In parallel, the effect of ANP on I_CaL_ was also examined in early myocytes isolated from mouse embryo heart (E8.5d). Similarly to EDS cardiomyocytes derived from ES cells, peak I_CaL_ amplitude was reduced by 65.4±8.6% by ANP in embryo heart cells tested (data not shown, n = 16).

### ANP Inhibits ISO Pre-Stimulated I_CaL_ in LDS Cardiomyocytes

**Figure 4:**
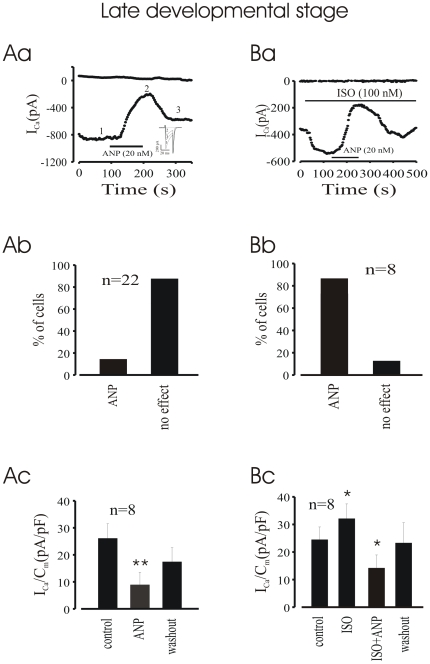
Effect of ANP on basal and ISO stimulated I_CaL_ in LDS cells. **Aa & Ba**, time courses of peak I_CaL_ recorded from single LDS cells. Both basal and ISO pre-stimulated I_CaL_ were strongly depressed by application of ANP. **Ab & Bb**, percentage of cells responding to ANP. Note that the percentage of responding cell in EDS is much lower compared to LDS cells. **Ac & Bc**, current density of peak I_CaL_. *P<0.05, **P<0.01 compared with control, respectively.

Next we tested whether ANP also regulated I_CaL_ in LDS cardiomyocytes. Although ANP also decreased basal I_CaL_ in LDS cells (Fig. 4Aa), the effect was much less frequent as compared to EDS cardiomyocytes, with only about 34% cells (n = 22) which responded to the peptide (Fig. 4Ab). However, when I_CaL_ was pre-stimulated by ISO (100 nM), adding ANP on top of ISO induced a pronounced inhibition of I_CaL_ (Fig. 4Ba) which occurred in about 70% cells (Fig. 4Bb) and averaged 58.6±18.6% inhibition (n = 8, Fig. 4Bc). This effect was reversible in most of cells upon washout (Fig. 4Ba & c).Thus, our data indicate that ANP also regulates I_CaL_ in LDS cells, but, unlike the EDS cells, its effect requires, or at least is more prominent after a pre-stimulation of the current by β-adrenergic receptor.

### ATP-γ-S Plus Forskolin Block the Depression of ANP on I_CaL_ in EDS Cardiomyocytes

**Figure 5:**
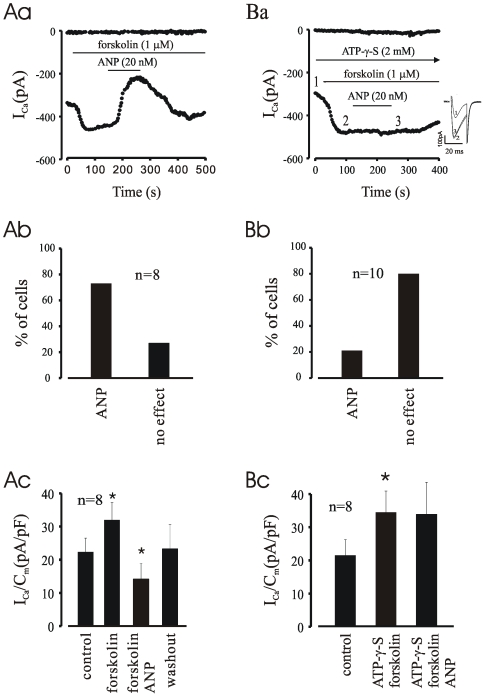
Effect of ATP-γ-S plus forskolin on ANP induced I_CaL_ inhibition. **Aa**, time course shows that ANP depressed forskolin pre-stimulated I_CaL,_ however the depressed effect of ANP on forskolin pre-stimulated I_CaL_ was abolished in the presence of ATP-γ-S (**Ba**). The insets are traces recorded in the same cells as time courses. **Ab & Bb**, percentage of cells with or without responding to ANP. **Ac & Bc**, density of peak I_CaL_. *P<0.05 compared with control.

The above results are reminiscent of the effect of the muscarinic agonist carbachol (CCh), which was reported in our earlier study to inhibit basal I_CaL_ in EDS cells but required ISO pre-stimulation of I_CaL_ to produce its inhibitory effect in LDS cells [27]. One possible difference between EDS and LDS cells is the higher intrinsic activation of the cAMP/PKA pathway in the former [27], pointing to a possible action of ANP on this pathway. In order to test this hypothesis, the effect of ANP was examined on EDS cells after pre-stimulation with the adenylyl cyclase activator forskolin (1 µM in bath solution) or ISO, and in the absence or presence of 2 mM ATP-γ-S in the patch pipette solution to block dephosphorylation. As shown in Fig. 5Aa, ANP decreased I_CaL_ stimulated by forskolin, but had no effect when ATP-γ-S was present in the patch-pipette (Fig. 5Ba). On average, the stimulatory effect of forskolin was not different in the absence (61.4±7.4%, n = 8) and presence of ATP-γ-S (63.6±8.6%, n = 12), but the effect was irreversible upon washout in the latter case. However, the effect of ANP on pre-stimulated I_CaL_ was abolished in almost all cells tested, suggesting that the effect of ANP on I_CaL_ was *via* inhibition of the cAMP/PKA axis.

### Role of G_i/o_ Proteins in the ANP-Mediated Inhibition of I_CaL_

**Figure 6:**
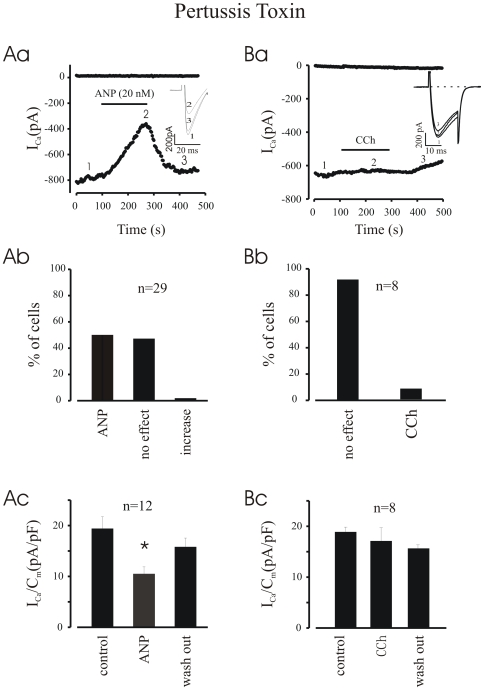
PTX did not abolish ANP induced I_CaL_ inhibition. **Aa**, time course demonstrates that in PTX pre-treated cardiomyocytes derived from ES cells ANP still inhibited I_CaL_. **Ba**, time course of PTX positive control **experiment** which indicates PTX abolished CCh caused inhibition on I_CaL_. **Ab and Bb**,percentage of cells with or without responding to ANP(Ab) or CCh (Bb). **Ac and Bc**, density of peak I_CaL_. *P<0.05 compared with control.

Since ANP blocks the cAMP/PKA pathway, its effect could be either through an inhibition of adenylyl cyclase *via* activation of pertussis toxin (PTX)-sensitive G_i/o_ proteins [29]–[31], or through cGMP generated by activation of the particulate GC. To test the former hypothesis, EDS cells were incubated with 1 µg/ml PTX for 12 hours. Under this condition, application of ANP still depressed I_CaL_ in 50% of both EDS (Fig. 6Aa and b) and LDS (data not shown) cells. Thus, the depression of I_CaL_ by ANP was not related to PTX sensitive G_i_/or G_o_ proteins. Fig. 6B shows a positive control for PTX in LDS cells.

### Role of cGMP-Activated PDE2 in the Inhibitory Effect of ANP on I_CaL_ in EDS Cardiomyocytes

**Figure 7:**
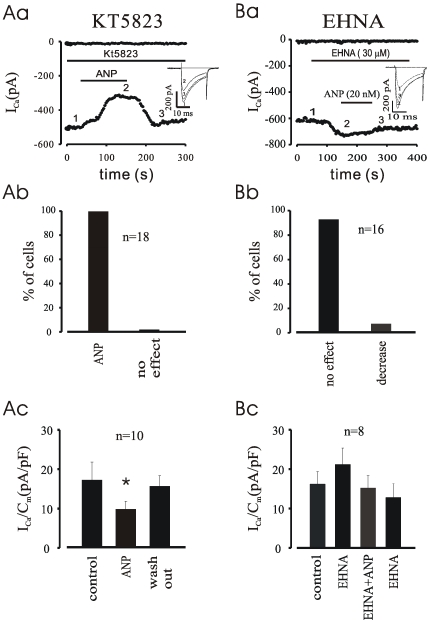
PDE2-cAMP-PKA pathway of ANP depression on I_CaL_. **A**, time course of I_CaL_ (a) shows ANP still inhibits the current in KT5823, a PKG selective inhibitor, pre-treated myocytes in about 70% cells tested (b); **Ac**, density of peak I_CaL._ **B**, time course (a) demonstrates that ANP fails to depress I_CaL_ in the presence of EHNA, a PDE2 selective inhibitor, in almost all cells tested (b). **Bc**, density of peak I_CaL._ *P<0.05 compared with control.

As shown earlier [27], the NO/soluble GC pathway is not involved in the inhibition of basal I_CaL_ in mouse EDS cells. However, since the ANP receptor activates the particulate GC, cGMP is a good candidate to mediate the inhibitory effect of ANP on basal I_Ca,L_. cGMP could inhibit I_CaL_ *via* either cGMP-dependent protein kinase (PKG) [32] or *via* activation of cAMP hydrolysis *via* stimulation of the cGMP-stimulated PDE2 [20], [33]. To discriminate between these two hypothesis, the effect of ANP on I_CaL_ was tested in the presence of the PKG inhibitor KT5823 or the PDE2 inhibitor EHNA. As shown in Figure 7A, KT5823 (1 µM) did not eliminate the effect of ANP in EDS cells tested. Conversely, EHNA (20 µM), which as shown earlier [27] increased the basal I_CaL_ (by 22.1±3.3%), blocked the ANP inhibitory effect on basal I_CaL_ in 94% of EDS cells tested (see also Fig. 7Ba, Bb, and Bc). Unlike EHNA, the PDE3 inhibitor milrinone (10 µM) did not block the inhibitory effect of ANP (20 nM), which still decreased I_CaL_ by 49.4±9.4% (data not shown). Taken together, these findings suggest that the decrease of I_CaL_ by ANP is mediated through an increase of cGMP levels, thereafter activation of cGMP-stimulated PDE2, enhancement of cAMP breakdown, decrease of PKA levels, and ultimately reduced phosphorylation of voltage dependent Ca^2+^ channels (VDCC) or a closely associated protein.

In order to prove that the effect of ANP on I_CaL_ is mediated by cGMP we examined the effect of 8-bromo-cGMP on I_CaL_ in early developmental cardiomyocytes derived from ES cells as well. As displayed in figure S2, upon application of 200 µM 8-bromo-cGMP led to a significant suppression of the L-type Ca^2+^ current and this effect of 8-bromo-cGMP was abolished by pre-treatment of the cells with EHNA. The experimental results of 8-bromo-cGMP confirmed that the depression of ANP on I_CaL_ was mediated by cGMP in early developmental cardiomyocytes.

## Discussion

Here, we report that ANP (3–28) exerts negative chronotropic effect without hyperpolarization in EDS cardiomyocytes derived from ES cells, EDS EB as well as the whole embryo heart. This is probably mediated by a depression of the basal I_CaL_ in EDS cardiomyocytes and in β-receptor pre-stimulated I_CaL_ in LDS cardiomyocytes because ANP did not exhibit any effect on both I_f_ and I_Ks_ currents in the present study (data not shown). From the time course of ANP action on I_CaL_ and APs we noted that when the I_CaL_ was reduced by about 30% of maximal effect by ANP the negative chronotropic effect occurred, and when the reduction of the I_CaL_ reached the maximum the APs were completely inhibited in almost all experiments conducted if washout was not followed (Fig. S1B). ANP depressed I_CaL_ without an effect on the I/V relationship, indicating that the degree of I_CaL_ inhibition was proportional at all potentials, and that the depressing effect of ANP on I_CaL_ was completely abolished by clamping cAMP or PKA levels which suggests that ANP is only acting through activation of the cGMP stimulated PDE2. Indeed, EHNA, the selective PDE2 inhibitor, resulted in a complete abolishment of ANP action on I_CaL_ implying that the inhibitory effect of ANP was through the cGMP/PDE2/cAMP pathway (Fig. 7B), and this was further proved by the results of 8-bromo-cGMP (supplemental Fig. S2). In the presence of L-NAME as well as of 1H-(1,2,4)oxadiazolo(4,3-a)quinoxalin-1-one (ODQ) [27], however, ANP-still decreased I_CaL_ excluding a role of NO or sGC.

The evidence that ANP has a direct effect on I_CaL_ in the heart is controversial. Most studies suggest that ANP has a direct effect only in the SA node in isolated atrial tissue preparations [12], [18], [34], [35]. Other reports support the notion that ANP has no direct effect on action potential duration (APD) or contractility of isolated guinea pig ventricular papillary muscle [36]–[38]. While ANP may not have a direct negative chronotropic effect, the receptor linkage to the guanylyl cyclase in rabbit atrium [39], rabbit [40] and rat ventricle [41], [42], as well as rabbit Purkinje fibers [43], [44] has been proven. Moreover, ANP receptor stimulation results in an increase of intracellular cGMP and thereafter a decrease of cAMP levels [14]. These findings are well in line with our results where the ANP effect in EDS cells was fully mediated by a PDE-II mediated decrease of cAMP levels. The negative chronotropic effect of ANP is in agreement with results reported by Favaretto-AL and co-workers [45].

ANP has been demonstrated to exert an effect on both basal I_CaL_ as well as pre-stimulated I_CaL._ In human atrial cells, 10 nM ANP reduces I_CaL_ [12] which suggests that ANP-induced inhibition of I_CaL_ was *via* a cGMP-dependent mechanism [19]. Similar results have been found in chicken embryonic myocytes [46] and in rabbit ventricular cells. Both cell types are characterized by high intrinsic adenylyl cyclase activities. In rabbit ventricular cells, ANP also produced a concentration-dependent decrease of basal I_CaL_. This effect was blocked upon application of 8-bromo-cGMP, a non-metabolizable analog of cGMP. Single channel recordings revealed that ANP reduces the open probability of Ca^2+^ channel without affecting the unitary conductance [18]. The catecholamine-stimulated I_CaL_ was also attenuated by ANP. In human cardiomyocytes, ANP reduced ISO-stimulated I_CaL_ by 25% through a cGMP-dependent mechanism [23]. Gisbert and Fischmeister [17] demonstrated that I_CaL_ was also inhibited by ANP in single frog ventricular myocytes. They suggested that ANP had a direct G-protein mediated effect on AC. However, we here demonstrate at least for the EDS cells that ANP action is still preserved in PTX incubated cells, but abolished after clamping cAMP levels.

It is known that ANP exerts its physiological and pathological role *via* an increase of cellular cGMP levels [18] due to specific activation of the particulate guanylyl cyclase [5]. Our previous data of ODQ as well as MB excluded an involvement of the soluble guanylyl-cyclase [27]. An involvement of PKG in the ANP-mediated depression of I_CaL_ was excluded for ES cell-derived cardiomyocytes since in the presence of KT5823, a PKG selective inhibitor, ANP still inhibited the I_CaL_ (Fig. 7A), and clamping of cAMP-PKA levels abolished I_CaL_ depression. This is in clear contrast to a report [18], where ANP decreased basal I_CaL_ through intracellular production of cGMP and activation of PKG in rabbit heart cells. Thus, we show that the depression of ANP on I_CaL_ is through the particulate GC, cGMP and PDE2 mediated depression of cAMP-PKA.

It has previously reported that another fragment of ANP, ANP1–28, also possesses inhibitory effect on I_CaL_ in rabbit heart cells [18]. In the present study we also examined this fragment in our models, and the results suggested that the inhibitory effect of ANP1–28 on I_CaL_ was weaker compared to that of ANP3–28. The reason for the discrepancy between 3–28 ANP and 1–28 ANP was unclear though the model difference might be one possible reason.

In conclusion, ANP depresses I_CaL_ via pGC/cGMP and PDE2 mediated cAMP/PKA breakdown pathway in early developmental cardiomyocytes.

## Supporting Information

Figure S1 Dose-response and inhibition of ANP on action potentials. A, concentration-response relation between ANP and the decrease in Ca^2+^ current. B, action potentials were almost completely inhibited by ANP (20 nM) if washout not followed. Data are mean ± s.e. mean. *P<0.05, **P<0.01.
(0.92 MB TIF)

Figure S2 Effects of 8-Bromo-cGMP on L-type Ca^2+^ current in EDS cardiomyocytes. Time course of ICaL demonstrates that 8-Bromo-cGMP (200 µM) depresses ICaL (A), and the effect of 8-Bromo-cGMP on ICaL is abolished by pre-treatment of the cells with EHNA, a specific inhibitor of PDE2 (B). C, percentage of cells responding to 8-Bromo-cGMP. D, density of peak ICaL. **P<0.01 compared with control.
(0.70 MB TIF)
