# Supplementary figures and images for: Atrial Natriuretic Peptide Regulates Ca2+ Channel in Early Developmental Cardiomyocytes
Source: PLoS One. 2010 Jan 22;5(1):e8847. doi: 10.1371/journal.pone.0008847 (PMC2809742; doi:10.1371/journal.pone.0008847)

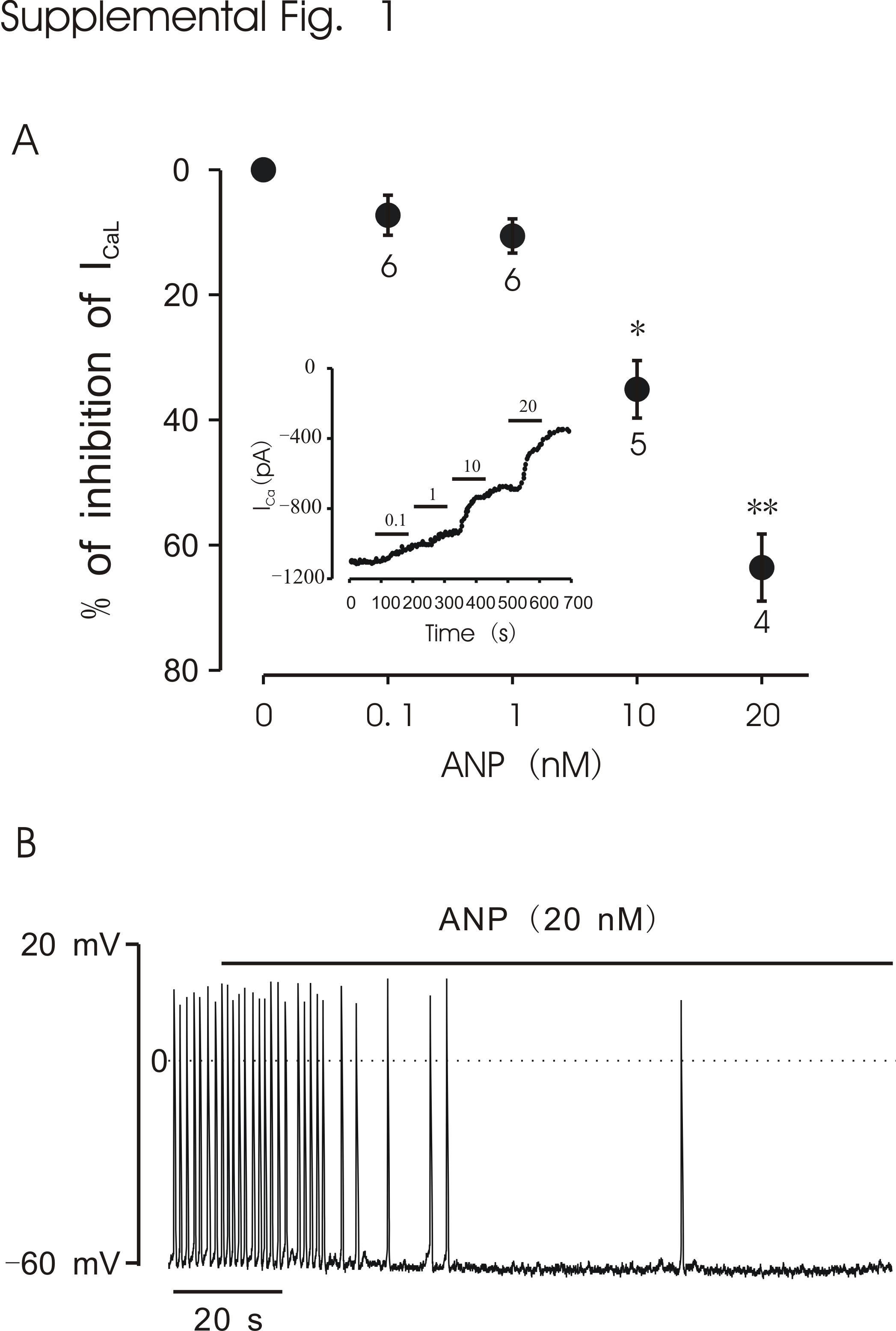

Supplement: Figure S1 — Dose-response and inhibition of ANP on action potentials. A, concentration-response relation between ANP and the decrease in Ca2+ current. B, action potentials were almost completely inhibited by ANP (20 nM) if washout not followed. Data are mean ± s.e. mean. *P<0.05, **P<0.01. (0.92 MB TIF) [file pone.0008847.s001.tif]

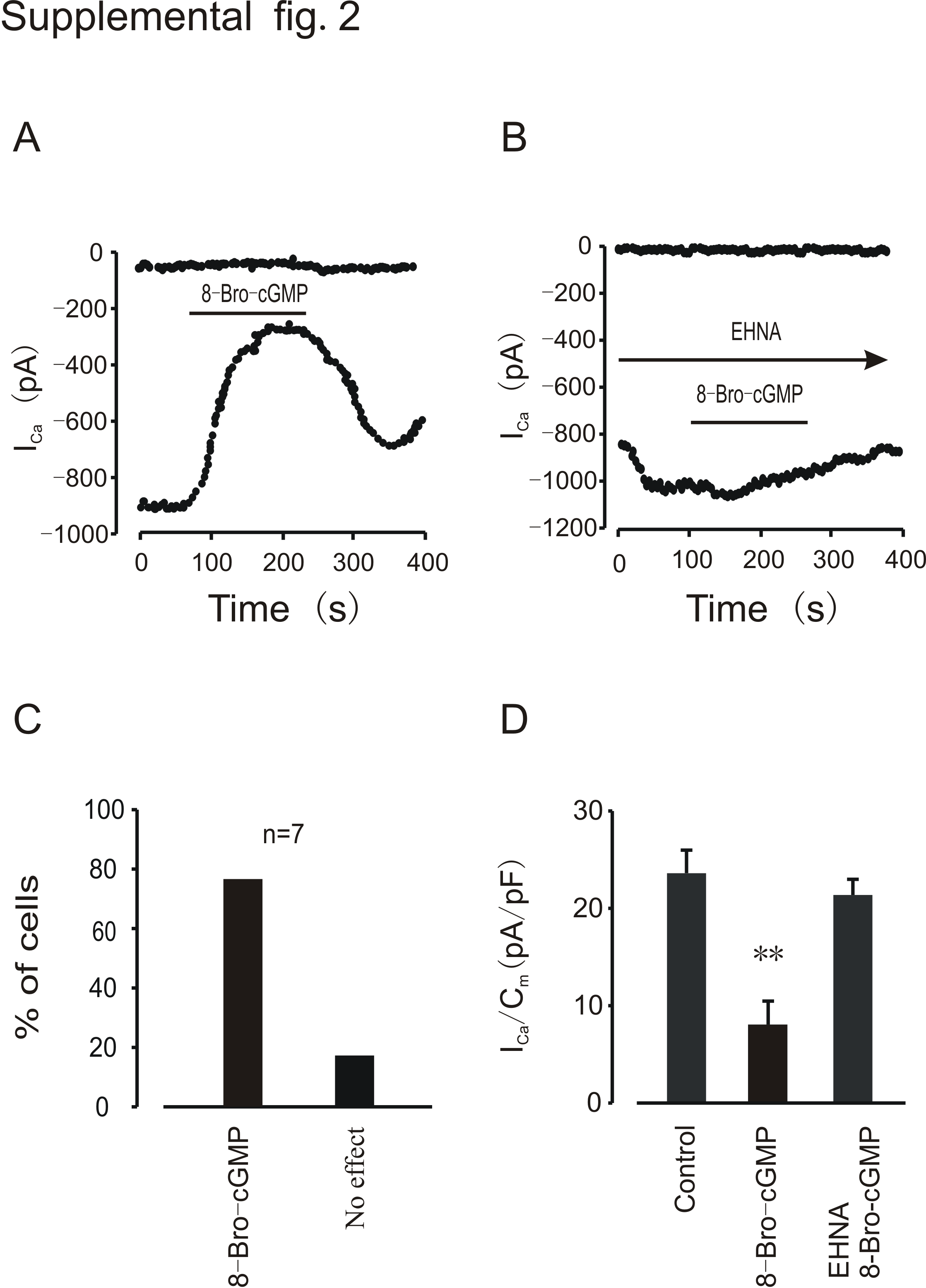

Supplement: Figure S2 — Effects of 8-Bromo-cGMP on L-type Ca2+ current in EDS cardiomyocytes. Time course of ICaL demonstrates that 8-Bromo-cGMP (200 µM) depresses ICaL (A), and the effect of 8-Bromo-cGMP on ICaL is abolished by pre-treatment of the cells with EHNA, a specific inhibitor of PDE2 (B). C, percentage of cells responding to 8-Bromo-cGMP. D, density of peak ICaL. **P<0.01 compared with control. (0.70 MB TIF) [file pone.0008847.s002.tif]
